# Co‐cultivation of diazotrophic terrestrial cyanobacteria and *Arabidopsis thaliana*

**DOI:** 10.1002/elsc.202000068

**Published:** 2020-11-23

**Authors:** Dorina Strieth, Sarah Di Nonno, Judith Stiefelmaier, Jonas Kollmen, Doris Geib, Roland Ulber

**Affiliations:** ^1^ Bioprocess Engineering Technical University Kaiserslautern Kaiserslautern Germany

**Keywords:** co‐cultivation, cyanobacteria, diazotrophic biofilms, phototrophic biofilms, plants

## Abstract

Diazotrophic cyanobacteria are able to fix N_2_ from the atmosphere and release it as bioavailable nitrogen what other organisms can utilize. Thus, they could be used as living nitrogen supplier whereby the use of fertilizer could be reduced in agricultural industry what results in a decrease of laughing gas released during fertilizer production. The diazotroph cyanobacterium *Desmonostoc muscorum* (*D. muscorum*) was characterized in shake flasks cultivated in nitrogen‐free and nitrogen‐containing medium. Similar growth rates were reached in both cultivations and the release of ammonium by *D. muscorum* was detected under nitrogen depletion. Subsequently, *D. muscorum* was co‐cultivated with *Arabidopsis thaliana* (*A. thaliana*) in nitrogen‐free medium. Additionally, the plant was cultivated in nitrogen containing and nitrogen‐free medium without *D. muscorum* as reference. A co‐cultivation led to higher growth rates of the cyanobacterium and similar growth of *A. thaliana* with similar maximum photochemical efficiency of photosystem II compared to the growth of nitrogen containing medium. Further, accumulation of cyanobacterial cells around the roots of *A. thaliana* was detected, indicating a successfully induced artificial symbiosis. Based on these results, *D. muscorum* could be a promising cyanobacterium as living nitrogen supplier for plants.

**Abbreviations**
*A. thaliana*: *Arabidopsis thaliana*
BG‐11: Blue‐Green Medium
BG‐11‐0: Blue‐Green Medium without nitrogen
CDW: cell dry weight
CWW: cell wet weight
EP: Sextracellular polymeric substances
MS: Murashige skoog medium
MS‐0: Murashige skoog medium without nitrogen
*D. muscorum*: *Desmonostoc muscorum*
PS II: photosystem II, n_b_ = biological replicate

## 1 INTRODUCTION

Today, agriculture is faced with major challenges due to a growing population and the resulting growing food demands. Because of that the inset of fertilizer, especially nitrogen and phosphorus fertilizer, is indispensable [1]. For this purpose, either organic material, in the form of humus or animal excrements or mineral nitrogen fertilizer, which consists of ammonium or nitrate salts, can be added. Here, the ammonification supplies ammonia and ammonium from the breakdown of organic matter and animal urea, while the bacterial nitrification by *Nitrosomonas* and *Nitrobacter oxidizes* ammonium via nitrite to nitrate. In this process, ammonia (NH_3_), nitrogen monoxide (NO), nitrogen dioxide (NO_2_, laughing gas) and elemental nitrogen (N_2_) evaporate. In particular, nitrous oxide is very inert in the troposphere and has an average residence time of 70 [2] to 150 years [3]. The CO_2_ equivalent of N_2_O is given as 310 ppm and nitrous oxide, thus, contributes significantly to the greenhouse effect. Cyanobacteria could offer an alternative for the inset of fertilizer because of their plant growth‐promoting abilities [4, 5, 6, 7]. Cyanobacteria are prokaryotes that can not only grow aquatic, but also terrestrial in the form of biofilms with surface association and air exposed [8, 9]. They are found in a diverse environment, for example deserts [10], hot springs [8] or the Antarctic [11, 12]. To make life possible under these conditions, in particular terrestrial cyanobacteria produce a matrix of extracellular substances that fulfill a wide variety of protective factors. They act for example as water and nutrient storage and enable to adhere to surfaces like rocks [13]. Cyanobacteria are capable of performing oxygenic photosynthesis. In addition to chlorophyll and carotenoids present in plants, cyanobacteria have other antenna pigments which, through the absorption of other wavelength ranges, allow more effective use of light. Through chromatic adaptation, they can adapt their pigment content to ambient conditions, which is why the pigment composition provides information about the status of the cells [13]. Furthermore, some of them are able to fix chemically inert nitrogen from the air [14]. Since oxygen inactivates nitrogenase [15] some cyanobacteria separate photosynthesis from N_2_‐fixation locally through formation of heterocysts [15]. Heterocysts are specialized for N_2_‐fixation for example through a thicker cell wall to prevent oxygen ingress and decrease in pigmentation caused by a lack of photosystem II (PS II) what is responsible for the splitting of water into oxygen and protons [16]. These attributes make them an important part of the carbon and nitrogen cycle. Because of that cyanobacteria can be found in symbiosis with plants where they provide fixed nitrogen in the form of ammonium [17]. In contrast to rhizobium‐legume symbiosis, cyanobacteria are more diverse and the selection of terrestrial host plants for the cyanobionts is wide [18]. Furthermore, they supply other nutrients like amino acids [19], phytohormones [20, 21, 22], vitamins [23], polypeptides and extracellular polymeric substances (EPS) [24] that act as antimicrobial substances [19, 21, 25]. Cyanobacteria are able to solubilize mineral phosphorus and thus, make it available for their symbiotic partner [26]. Moreover, they improve the soil's water retention capacity and prevent eutrophication which enhances plant growth [19]. Positive effects of plant growth due to cyanobacterial inoculation have been reported for crops like rice [27, 28], maize [29], cotton [30], wheat [6, 7], peas [31] and tomatoes [32, 33, 34].

In this work, the diazotrophic cyanobacterium *Desmonostoc muscorum* is cultivated in shake flasks with nitrogen‐free and nitrogen‐containing medium. Thereby, the influence of nitrogen deficiency in the medium on growth, EPS content, pigment and phycobiliprotein composition and the composition of the supernatant is investigated. Furthermore, it is examined wether *D. muscorum* enters into a symbiosis with the model plant *A. thaliana*. As reference *A. thaliana* is cultivated in standard nitrogen‐containing medium and as axenic culture in nitrogen‐free medium.

1 **PRACTICAL APPLICATION**

Co‐cultivation of diazotrophic cyanobacteria and plants can lead to a decrease of fertilizer consumption resulting in a reduced release of laughing gas during fertilizer production. *Desmonostoc muscorum* (*D. muscorum*) was characterized in nitrogen‐containing and nitrogen‐free medium. Similar growth rates were detected, and ammonium was released by *D. muscorum* starting in exponential phase, correlating with formation of heterocysts under nitrogen limitation. The combination of similar growth rates and ammonium release into the environment speaks for a good nitrogen‐fixer. A co‐cultivation of *D. muscorum* and *Arabidopsis thaliana* (*A. thaliana*) was successful, and plants looked healthier than cultivated under nitrogen limitation without *D. muscorum*. An accumulation of cyanobacterial cells around the roots of the plant could be detected what indicates for an artificial induced symbiosis. This study can form the basis for the use of *D. muscorum* as natural nitrogen‐fixer in the agricultural industry to reduce laughing gas in the atmosphere and to enhance plant growth.

## 2 MATERIALS AND METHODS

### 2.1 Pre‐culture

In this study, the terrestrial cyanobacterium *D. muscorum* (90.3) was used. *D. muscorum* was provided by Prof. Dr. Burkhard Büdel (Department of Plant Ecology and Systematics, University of Kaiserslautern, Germany) and collected from the soil in Columbia, USA. Pre‐cultures were cultivated in 300 mL shake flasks without baffles containing 50 mL of standard Blue‐Green medium (BG‐11) medium [35]. For incubation, a shaking incubator (Multitron S‐000115689, Infors HT, Bottmingen, Switzerland) at 120 rpm with 2.5 cm eccentricity was used, with a constant temperature of 30°C and continuous lightning at 100 μmol_photons_ m^−2^ s^−1^. The radiometer LI‐1400 equipped with a quantum sensor 190A (LI‐COR Biosciences, Lincoln, USA) was used to adjust the light intensity. Pre‐cultures were harvested after 2 weeks of cultivation. Cell suspension was transferred into a 50 mL plastic reaction vessel and centrifuged for 15 min at 8000 g (centrifuge 383 K, Hermle Labortechnik GmbH, Wehingen, Germany). The supernatant was discarded, and the biomass pellet was used for further experiments.

### 2.2 Cultivation and characterization of *D. muscorum* in shaking flasks

Since characterization of phototrophic biofilms on agar plates is challenging, *D. muscorum* was characterized in shaking flasks using medium with (BG‐11) and without nitrogen (BG‐11‐0). 24 shaking flasks (300 mL without baffles) were inoculated with 0.1 g cell wet weight (CWW) and 50 mL of medium. After 1, 2, 3, 4, 7, 9, 11, and 14 days three shaking flasks were picked, cell dry weight (CDW) was determined and EPS, phycobiliproteins and pigments were extracted and analyzed according to the combined extraction strategy described by Strieth and Stiefelmaier et al. [36]. Additionally, components of the medium were quantified by compact ion exchange chromatography with inline system for dialysis (930 Compact IC Flex, Metrohm, Filderstadt, Germany) with a conductivity detector over the cultivation period. Anions (chloride, nitrite, nitrate, phosphate and sulfate) were measured with an anion column (Metrosep A Supp 5–250/4.0, Metrohm) using 1 mM NHCO_3_ and 3.2 mM NA_2_CO_3_ as mobile phase at a flow rate of 0.7 mL min^−1^. Cations (ammonium, magnesium, sodium, potassium) were measured with a cation column (Metrosep C6‐250/4.0, Metrohm) using 4 mM nitric acid and 0.7 mM dipicolin acid at a flow rate of 0.9 mL min^−1^. In both cases, the oven temperature was set to 35°C

### 2.3 Characterization of cyanobacterial growth on agar plates

For plant cultivation, in most cases the MurashigeSkoog medium (MS) in half strength is used [37]. Potassium nitrate and ammonium nitrate have not been added to prepare the nitrogen free MS‐0 medium. Since for cyanobacteria BG‐11 medium is the standard medium the influence of MS and BG‐11 medium on cyanobacterial growth was investigated. Additionally, the influence of nitrogen free medium MS‐ medium (MS‐0) and nitrogen free BG‐11 medium (BG‐11‐0) on growth of *D. muscorum* was investigated. Agar plates (Ø 5 cm) were prepared using the media MS/MS‐0 and BG‐11/BG‐11‐0 by adding 0.8% plant agar. Plates were inoculated with centrifuged CWW by a small dot of CWW. Growth was determined non‐invasive by detecting the chlorophyll‐a fluorescence using PAM fluorometry (IMAGING‐PAM‐Series, Walz GmbH, Effeltrich, Germany) as indicator to determine areal growth according to Stiefelmaier and Strieth et al. [38]. Since start concentration of biomass was different, percentage growth was calculated for a better comparison. Cyanobacteria were cultivated at 26°C in a climatic cabinet, the illumination was carried out for 16 h day^‐1^ with a photon flux of approx. 90 μmol_photons_ m^−2^ s^−1^.

### 2.4 Experimental set‐up of co‐cultivation

**FIGURE 1:**
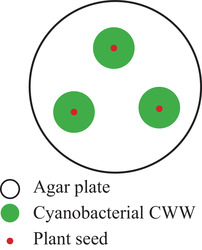
Experimental set‐up of co‐cultivation. Agar plates were inoculated with three dots of cyanobacterial Cell Wet Weight (CWW). In each dot one sterilized plant seed was placed

For co‐cultivation, agar plates with MS‐0 with 0.8% plant agar were used. The agar plates were inoculated with three dots containing 0.1 g cyanobacterial CWW on 250 mm^2^ (n_b_ = 3 per cyanobacterial strain). In each dot one surface sterilized seed of *A. thaliana* was placed (see Figure 1). The surface sterilization was carried out by a single rinse with 70% ethanol solution, followed by a 20‐min wash in a 10% sodium hypochlorite solution with 0.1% Triton‐X‐100 and finally rinsing with distilled water. To completely remove the sodium hypochlorite solution, seeds were rinsed with water several times. Additionally, seeds of *A. thaliana* were sown on agar plates with MS‐0 medium without *D. muscorum* as negative control. After inoculation of the plates a 3‐day vernalization followed. Therefore, the plates were stored in the dark at 4°C. Afterwards, the organisms were cultivated at 26°C in a climatic cabinet, the illumination was carried out for 16 h day^‐1^ with a photon flux of approx. 90 μmol_photons _m^−2^s^−1^. At regular intervals, the areal growth of the cyanobacteria was recorded by imaging PAM [38] and the root growth of the plants by microscopic (Nikon Eclipse Ni, Nikon, Minato, Shinagawa, Tokyo, Japan) imaging.

### 2.5 Determination of photosynthetic efficiency over chlorophyll fluorescence of *A. thaliana*

PAM‐fluorometer was used to determine the quantum yield of *A. thaliana*. Therefore, plants were dark‐adapted for 15 min and maximal photosystem (PS) II quantum yield was determined according to the following equation:

(1) $$\frac{F_{v}}{F_{m}^{{}^{\prime }}}=\frac{\left(F_{m}^{{}^{\prime }}-F_{0}\right)}{F_{m}^{{}^{\prime }}}\;$$

Where F_m_^′^ [‐] is the maximum fluorescence yield, F_v_ [‐] is the variable fluorescence and F_0_ [‐] is the dark fluorescence yield. After dark adaptation all PS II reaction centers are open and non‐photochemical energy dissipation is minimal, whereby maximal fluorescence yield (F_m_) is reached during a saturation pulse.

## 3 RESULTS AND DISCUSSION

### 3.1 Characterization of *D. muscorum* in shake flasks with and without nitrogen

**FIGURE 2:**
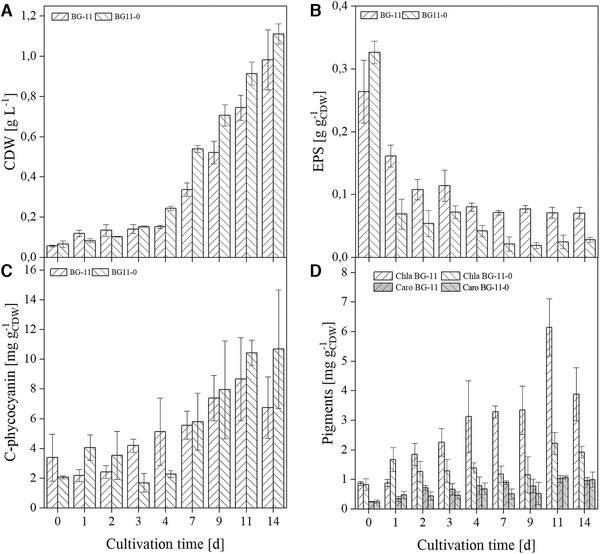
Characterization of *D. muscorum* in BG‐11 and nitrogen free BG‐11‐0 medium in shake flasks over the cultivation time. (A) Cell dry weight (CDW) per liter. (B) EPS content per CDW. (C) C‐phycocyanin content per CDW. (D) Content of chlorophyll‐a (chla) and carotenoids (caro) per CDW. Cultivation parameters: cultivation time = 14 days, continuous illumination with a light intensity = 100 µmol_photons_·m ^−2^·s^− 1^, temperature = 30°C, BG‐11, respectively BG‐11‐0 (nitrogen free), 300 mL shake flasks without baffles, n_b _= 3

**FIGURE 3:**
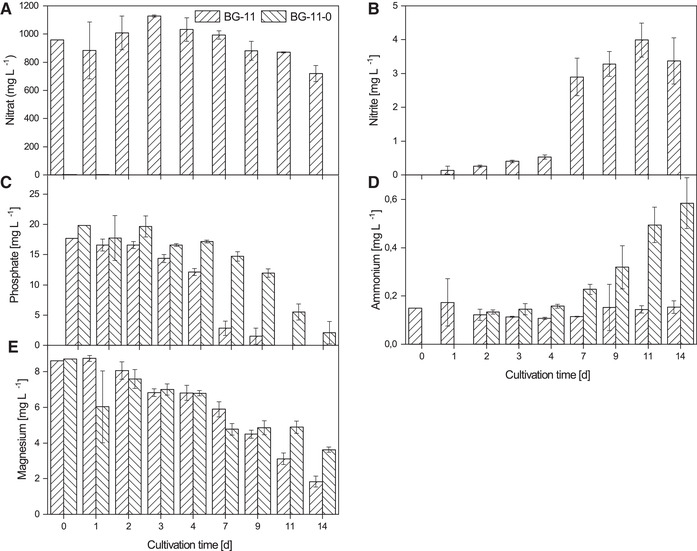
Characterization of the most important media components of *D. muscorum* cultivations in BG‐11 and nitrogen free BG‐11‐0 in shake flasks over the cultivation time. (A) Nitrate, (B) Nitrite, (C) Phosphate, (D) Ammonium, (E) Magnesium. Cations and anions were determined with ion exchange chromatography. Cultivation parameters: cultivation time = 14 days, continuous illumination with a light intensity = 100 µmol_photons_·m ^−2^·s^− 1^, temperature = 30°C, BG‐11, respectively BG‐11‐0 (nitrogen free), 300 mL shake flasks without baffles, n_b _= 3

**FIGURE 4:**
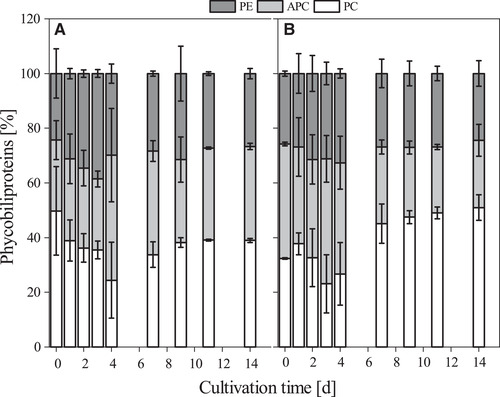
Composition of phycobiliproteins over the cultivation time of *D. muscorum* cultivated in BG‐11 (A) and nitrogen free BG‐11‐0 (B) medium. Phycobiliprotein composition is given in percent. PE = Phycoerythrin. APC = Allophycphycocyanin. PC = C‐phycocyanin. Cultivation parameters: cultivation time = 14 days, continuous illumination with a light intensity = 100 µmol_photons_·m ^−2^·s^− 1^, temperature = 30°C, BG‐11, respectively BG‐11‐0 (nitrogen free), 300 mL shake flasks without baffles, n_b _= 3

In the first step, it was investigated if the cyanobacterial strain *D. muscorum* has the ability to fix atmospheric nitrogen. Therefore, the cyanobacterium was grown in shaking flasks with and without nitrate and growth was recorded. In the literature mainly reduced growth under nitrogen limitation is described [39, 40]. Contrary to the expectations, no differences in growth could be detected with and without nitrate for *D. muscorum* (see Figure 2A). It was also expected that the lag phase would be longer under nitrogen‐limited conditions than under standard conditions, as the cells first have to adapt to the new conditions. Interestingly, the lag phases with and without nitrogen were of equal length. When analysing the supernatant, it was noticeable that *D. muscorum* absorbed only small amount of nitrate from the medium during the first 4 days of cultivation cultivated with BG‐11. Nitrogen uptake then occurred in the beginning of exponential phase and correlates with nitrite release in the supernatant (see Figure 3A,B) which is an intermediate product in nitrate assimilation [41]. No literature could be found on how nitrite is released into the medium. Only the uptake of nitrite is described [42]. Ammonium concentration was constant over the cultivation time (see Figure 3D). In contrast, no nitrite was released by cyanobacteria cultivated in nitrogen free medium, but ammonium concentration increased after the lag phase (see Figure 3B,D). Further, phosphate uptake of *D. muscorum* increased after lag‐phase and was lower when cultivated in nitrogen‐free medium compared to standard medium. Aubriot et al. reported that phosphate uptake was low under nitrogen depletion for non‐N_2_‐fixing, bloom‐forming cyanobacteria [43]. After addition of nitrate phosphate uptake was activated. For N_2_‐fixing *Dolichospermum flos‐aquae* it is shown that addition of nitrogen upregulates the genes involved in phosphate metabolism [44]. There seems to be a mutual nutrient dependence influenced by many different factors that need further research. Increasing ammonium concentration in the supernatant can be explained by heterocysts that were found after 4 days of cultivation (data not shown) in nitrogen free medium. Mostly 5 to 10% of vegetative cells differentiate to heterocysts in case of nitrogen deficiency in the medium [45]. In field studies, the nitrogen increase is usually explained by the death of cells through viral lysis [46], or programmed cell death [47], as the release of nitrogen does not seem to be useful for the cells. Despite the high energy cost of N_2_‐fixation, *Trichodesmium* sp. releases up to 80% of gross nitrogen fixation what indicates an active release [48]. Other diazotrophic cyanobacteria release between 40 and 80% dissolved bioavailable nitrogen of the N_2_‐fixation rate [49, 50]. At first sight this does not seem to make sense, since the energetic costs of N_2_‐fixation are high compared to the assimilation of nitrate. Different explanations for the release of bioavailable nitrogen can be found in literature like nitrogen supply for vegetative cells lacking the enzyme nitrogenase in form of NH_4_/NH_3_ [48] or the excretion of amino acids like glutamine [16, 51]. The 4 days lag‐phase in nitrogen‐free and standard BG‐11 medium can be explained by the consumption of internal and external nitrogen stores. That could also explain the late formation of heterocysts that is described in literature after 24 h for *D. muscorum* [52]. The EPS content in both cases decreased over the cultivation time whereby the decrease was higher under nitrogen limitation (see Figure 2B). Khani et al. reported that different nitrogen sources have an impact on EPS formation of *Chryseobacterium indologenes* MUT.2 [53]. Pannard et al. described a decreased EPS production under nitrogen limitation of *Microcystis aeruginosa* [54]. Additionally, tryptophan‐like substances were found in EPS of *Microcystis* [55] that can act as an external nitrogen storage. *D. muscorum* is expected to deplete the internal and external nitrogen stores during the switch to diazotrophic growth, which is reflected in a reduction in EPS. This theory is supported by the decreasing C‐phycocyanin content in the lag phase under nitrogen limited growth (see Figure 2C). In both cultivations C‐phycocyanin was built up in the same way, but the composition of phycobiliproteins was different (see Figure 4). The phycobiliproteins showed a constant ratio of 1:1:1 (phycocyanin: allophycocyanin: phycoerythrin) over the cultivation time when cyanobacteria were cultivated in BG‐11. In contrast, the ratio of phycobiliproteins changed over the cultivation time when *D. muscorum* was cultivated in BG‐11‐0 from 1:1:1 to 2:1:1 (C‐phycocyanin: allophycocyanin: phycoerythrin). Liotenberg et al. described that overall phycobilisome number and rod length are similar in nitrate ‐and ammonium‐grown cells whereby specific composition of the rods is modified [56]. Carotenoid content was constant over the cultivation period when cultivating *ND muscorum* with and without nitrogen in the medium (see Figure 2D). That was expected since carotenoids inter alia act as cell wall stabiliser and UV protector and cultivation conditions should not lead to abiotic stress. Chlorophyll‐a content increased over the cultivation time in both cultivations, but higher concentrations were reached in BG‐11 (see Figure 2D). This correlates with magnesium uptake by *D. muscorum* that is higher in BG‐11 compared to BG‐11‐0 (see Figure 3E). Zhao et al. investigated the impact of nitrogen starvation on photosynthetic performance of *Porphyridium cruentum*. Thereby, chlorophyll content decreased under nitrogen starvation and maximum photochemical efficiency of PS II decreased during initial period of nitrogen deficiency [57]. In this study a constant chlorophyll content of *D. muscorum* was detected as well as identical growth rates when cultivated in BG‐11 (0.31 ± 0.01 d^−1^) and BG‐11‐0 (0.29 ± 0.02 d^−1^). That is why a reduction of maximum photochemical efficiency of PS II can be neglected. Additionally, *P. cruentum* is a non‐diazotrophic organism and can, thus, not adapt to N_2_‐fixation. Maybe the lower chlorophyll‐a‐content of *D. muscorum* can partly be explained by the differentiation of vegetative cells to heterocysts that are not capable of photosynthesis because oxygen inactivates the nitrogenase and thus N_2_‐fixation [15]. Before co‐cultivations of *D. muscorum* and *A. thaliana* were started it was investigated whether the cyanobacterium could be cultivated on the Murashige skoog medium (MS) optimized for plants. Therefore, the growth of *D. muscorum* was examined on agar plates using BG‐11 and MS medium with and without nitrogen. Maximum growth rates were determined over areal growth after Stiefelmaier and Strieth et al. [38]. Maximum growth rates were similar on BG‐11 (0.15 ± 0.03 d^−1^), on BG‐11‐0 (0.12 ± 0.03 d^−1^), on MS (0.13 ± 0.03 d^−1^) and on MS‐0 (0.13 ± 0.2 d^−1^). It can be assumed that the MS medium has no negative influence on the growth of *D. muscorum*. Additionally, as in submerged cultivation in shake flasks, the growth of *D. muscorum* was identical in nitrogen‐free and nitrogen‐containing medium.

### 3.2 Co‐cultivation of *D. muscorum* and *A. thaliana*

**FIGURE 5:**
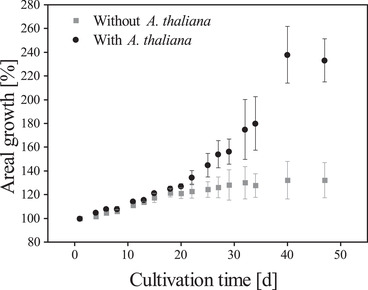
Areal growth of *D. muscorum* with and without *A. thaliana* on agar plates without nitrogen. Cultivation parameter: Cultivation time = 47 days, Light intensity = 90 µmol_photons_ m ^−2^ s^−1^; Day/Night Rhythm = 16/8 h; Temperature = 26°C, Murashige skoog medium without nitrogen (MS‐0). Areal growth was determined using PAM fluorometry, n_b_ = 3

**FIGURE 6:**
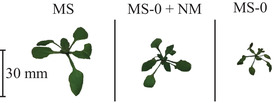
Pictures of *A. thaliana* cultivated 14 days in MS and MS‐0 and in co‐culture with *D. muscorum* in MS‐0. Cultivation parameter: Cultivation time = 47 days, Light intensity = 90 µmol_photons_ m ^−2^ s^−1^; Day/Night Rhythm = 16/8 h; Temperature = 26°C

**FIGURE 7:**
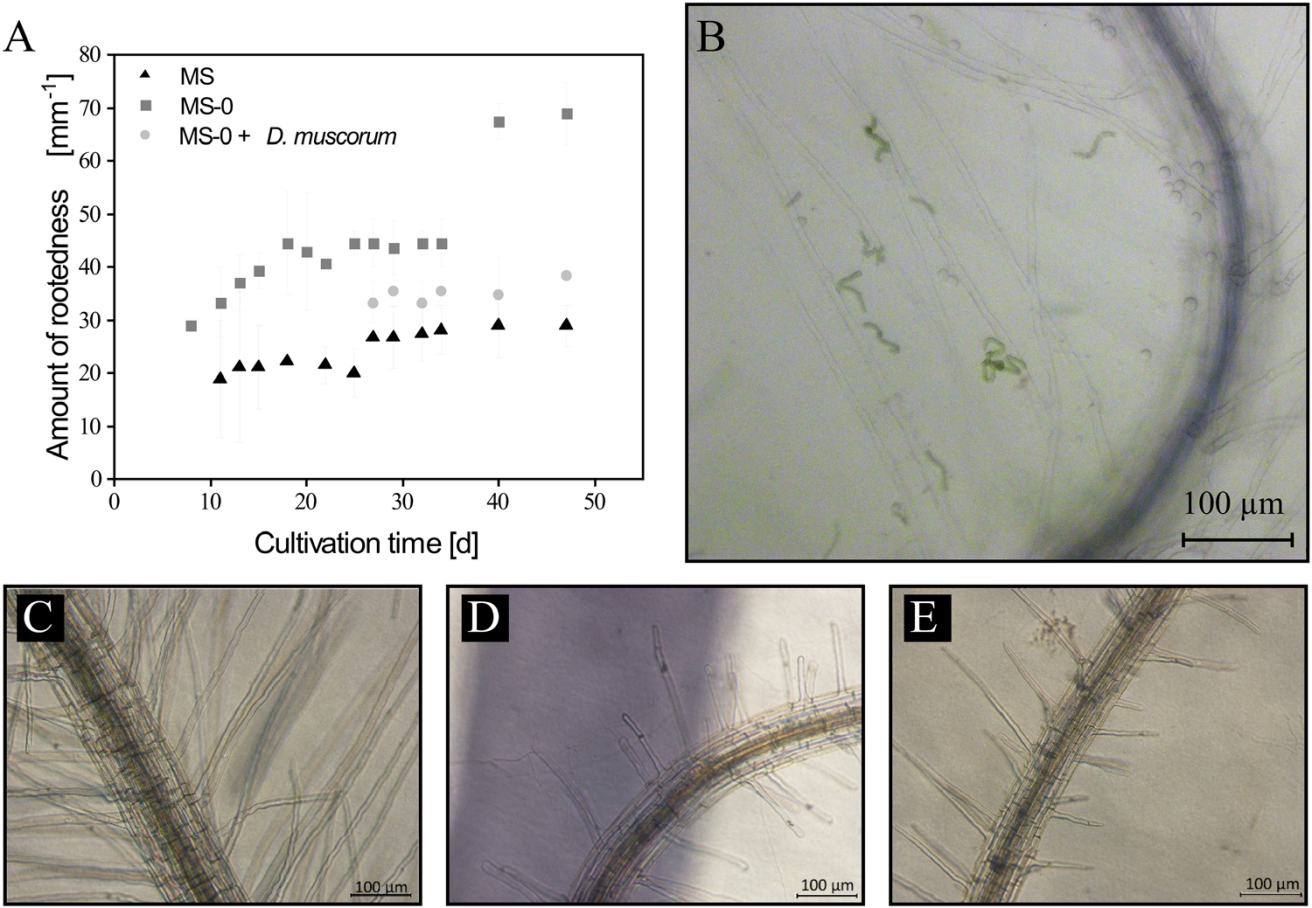
Root formation of *A. thaliana* in MS medium, nitrogen free medium (MS‐2) and MS‐0‐medium with *D. muscorum*. (A) Amount of rootedness of *A. thaliana* under different growth conditions over the cultivation period. (B) Microscopic image of *D. muscorum* on roots of *A. thaliana* after 47 days of cultivation. (C‐E) Microscopic images of rootedness of *A. thaliana* after 14 days of cultivation. (C) Roots of *A. thaliana* on MS‐0‐medium, (D) on MS‐0 medium in co‐cultivation with *D. muscorum* and (E) on MS‐medium. Cultivation parameter: Cultivation time = 47 days, Light intensity = 90 µmol_photons_ m ^−2^ s^−1^; Day/Night Rhythm = 16/8 h; Temperature = 26°C, on MS, MS‐0 medium with 0.8% plant agar, n_b_ = 3, microscopic evaluation n = 7

Subsequently, *D. muscorum* was co‐cultivated on MS‐0 agar plates with *A. thaliana* and additionally as reference without *A. thaliana*. Furthermore, *A. thaliana* was cultivated on MS and on MS‐0 medium without cyanobacteria. *N. musocrum* showed enhanced growth (see Figure 5) and biomass turned dark green in co‐culture (data not shown). This behaviour could indicate the production of growth hormones from *D. muscorum* and is already described in literature [58, 59]. The release of growth hormones would be an indication of existing symbiotic relationships between the organisms. *A. thaliana* co‐cultivated with *D. muscorum* looked healthy and grew beyond the cotyledon stage (see Figure 6). The plants were minimally smaller and significantly higher than those cultivated on MS‐0 without *D. muscorum*. Additionally, maximum quantum yield of PS II was measured of *A. thaliana* over the cultivation period and was constant over the cultivation period at 0.88 ± 0.05 (MS), 0.71 ± 0.02 (MS‐0) and at 0.86 ± 0.01 (co‐cultivated with *D. muscorum* in MS‐O). The quantum yield is typically less than one and can be used for any defined light‐dependent process. It describes the rate of photon absorption by the system and thus gives the efficiency of photosynthesis [60]. In this case it can be seen, that photosynthesis efficiency of *A. thaliana* is similar when grown on MS and in co‐culture with *D. muscorum* on MS‐0 what indicates a good nutrient supply and thus a successfully artificial induced symbiosis. Since preliminary tests showed that the root morphology of *A. thaliana* differs greatly under different cultivation conditions [61, 62]. The root formation of *A. thaliana* was determined in co‐culture with *D. muscorum* using MS‐0‐medium and as axenic culture using MS‐0‐ and MS‐medium. Different root growth of *A. thaliana* cultivated axenically on agar plates with and without nitrogen was detected (see Figure 7A, C, and E). On plates with no nitrogen and without symbiont, root growth was mainly horizontal. In addition, an increased expression of secondary roots was detected, indicating a limitation of nutrients, in this case nitrogen [62]. The number of lateral roots was significantly higher build by *A. thaliana* grown axenic on MS‐0 than in co‐culture with *D. muscorum*. Roots formed by axenic cultivation of *A. thaliana* were formed after 8 days of cultivation and increased within 10 days from 28.89 roots µm^−1^ to 44.44 ± 9.69 roots µm^−1^. The maximum number of roots was reached at the end of cultivation with 68.89 ± 5.88 roots µm^−1^. During cultivation with *D. muscorum* the first visible roots were formed after 27 days. Roots formed by co‐cultivated *A. thaliana* with *D. muscorum* were first visible after 27 days of cultivation. The amounts of lateral roots increased within 10 days only from 33.33 to 34.81 ± 7.14 roots µm^−1^ and stayed almost constant until the end of cultivation. Thus, the amounts of secondary roots formed by *A. thaliana* cultivated without cyanobionts was 44.09% higher than co‐cultivated with *N. muscorum*. This indicates a better nutrient supply of the plant with cyanobionts [62]. Especially the availability of phosphate and nitrogen influences the root system in different ways. In most cases, poor nutrient availability stimulates the growth of the secondary roots [61, 62]. A lack of nitrate often causes an increase in the length of the lateral roots [61, 62]. After 47 days; however, microscopic images confirmed the accumulation of cyanobacteria around the roots of the plant (see Figure 7B) for which cyanobacteria had to slide actively through the agar. This accumulation around and in the roots was already reported by Lindblad in Cyanobacteria‐Zamia symbioses [63, 64].

## 4 CONCLUDING REMARKS

In this is study it was investigated if *D. muscorum* can be used as living fertilizer for plants. Therefore, *D. muscorum* was characterized in nitrogen‐free and nitrogen‐containing standard medium in shake flasks. Similar growth rates were reached in both cultivations what does not correlate with the literature were mainly reduced growth is reported of diazotrophic organisms in nitrogen‐free medium. Additionally, formation of heterocysts is described for *D. muscorum* after 24 h and was microscopically detected after 3 to 4 days in this study. It was assumed, that *D. muscorum* deplete the internal and external nitrogen stores during the switch to diazotrophic growth, what can be supported by the reduction in EPS. Further, with appearing of heterocysts ammonium was released into the supernatant. Interestingly, the relation of phycobiliproteins stayed constant in reference cultivation and changed from 1:1:1 (C‐phycocyanin: allophycocyanin: phycoerythrin) to 2:1:1cultivated in nitrogen‐free medium. A nitrogen source dependent composition of phycobiliproteins is reported in literature. Since the cultivation of *D. muscorum* in nitrogen‐free medium was successful and bioavailable nitrogen was released into the environment, a co‐cultivation with *A. thaliana* was investigated. Therefore, the organisms were cultivated on nitrogen‐free (MS‐0) as co‐culture as well as s axenic culture on nitrogen‐containing (MS) and MS‐0 medium. The growth of *D. muscorum* was increased by the presence of the plant, due to the release of hormones which correlates with latest literature. Further, similar growth of *A. thaliana* with similar maximum photochemical efficiency of PS II compared to the growth of nitrogen containing medium was detected in co‐culture. An accumulation of cyanobacterial cells around the roots of *A. thaliana* were observed, indicating a successfully induced artificial symbiosis. In further research, co‐culture should be investigated on sand or in soil to investigate the application in agricultural industry. Additionally, long‐term studies over the seasons should be done to investigate productivities and the release of bioavailable nitrogen into the environment by the phototrophic biofilm. Based on the demonstrated results, *D. muscorum* could be a promising cyanobacterium as living nitrogen supplier for plants.

## CONFLICT OF INTEREST

The authors have declared no conflicts of interest.

## Nomenclature

**Table:** 

F_m_^′^	[‐]	maximum fluorescence yield
F_v_	[‐]	variable fluorescence yield
F_0_	[‐]	dark fluorescence yield

## Data Availability

Research data are not shared.
